# Atrial fibrillation in a child with COVID-19 infection

**DOI:** 10.1017/S1047951120003893

**Published:** 2020-10-19

**Authors:** Aura Daniella Santi, Paolo Aquino, Molly Dorfman

**Affiliations:** 1 Pediatrics Residency, Valley Children’s Hospital, Madera, CA 93636, USA; 2 Pediatric Cardiology, Valley Children’s Hospital, Madera, CA 93636, USA; 3 Pediatric Critical Care Medicine, Valley Children’s Hospital, Madera, CA 93636, USA

**Keywords:** multi-system inflammatory syndrome in children, COVID-19, cardiac arrhythmia

## Abstract

The SARS-CoV-2 (COVID-19) pandemic has challenged our initial predictions of its ramifications, both short and long term. Cardiovascular manifestations of COVID-19 in children remain a topic of investigation as literature is lacking. We describe new onset atrial fibrillation in a child with a history of COVID-19 infection. Understanding of cardiogenic effects of COVID-19 can help minimise the delay in diagnosis.

Cardiovascular manifestations of SARS-CoV-2 in children remain a topic of novel investigation as literature in this population is lacking. The adult population has been the primary focus for complications of this virus, and data are limited regarding cardiovascular manifestations in the paediatric population. A multi-system inflammatory syndrome in children has been described in association with COVID-19 infection, with 80% involving cardiovascular sequelae.^1^ Immunologic response appears to play a role in hyper-inflammation and cardiac injury.^2^ The mechanisms for cardiac injury have been proposed to be secondary to stress, with inflammatory responses leading to cardiac injury, and possibly cardiac muscle ischaemia.^3^ Cardiac manifestations of COVID-19 in adults to date include myocardial infarction, myocarditis, and cardiac arrhythmias.^3^ New onset arrhythmias are a poorly described complication in previously healthy children with COVID-19.^4^

## Case

A previously healthy, 17-year-old 86.3 kg male presented due to refractory hypotension requiring vasopressor support. Initially, the patient reported loss of smell and taste about 4 weeks prior to presentation. Two weeks prior to admission, the patient was noted to be COVID-19 positive, with intermittent fevers, progressively worsening cough, and shortness of breath, as well as an evanescent rash. Following a two-week quarantine, symptoms resolved until presentation, when the patient developed neck tenderness, vomiting, diarrhoea, and syncope. The patient was seen at an emergency department on multiple occasions for symptoms and discharged home with the diagnosis of COVID-19 and given Azithromycin for an unclear diagnosis, without resolution of symptoms. During the 24 hours prior to admission, the patient endorsed blurry vision, eye redness, chest pain, nausea, and vomiting. En route to the hospital, the patient complained of dizziness and reported syncope. On arrival, the patient was hypotensive to the 80s/40s. On initial physical exam, the patient was ill-appearing, with no significant respiratory impairment, pale and diaphoretic, tachycardic to 120s with sinus rhythm, and had a hyper-dynamic precordium. Mild hepatomegaly was noted. The conjunctiva was injected and tongue erythematous, and a blanching rash was noted on palms and wrists. Criteria were met for severe multi-system inflammatory syndrome in children given the cardiovascular, renal, gastrointestinal, mucocutaneous, and pulmonary involvement with history of COVID-19 infection.

He received a 3 L normal saline bolus, but continued to be hypotensive despite norepinephrine, before quickly changed to epinephrine for concerns of cardiogenic shock. Electrocardiogram at presentation showed sinus tachycardia. A blood gas was notable for respiratory acidosis with mild metabolic compensation and lactic acidosis, troponin and brain natriuretic peptide were elevated; he had evidence of acute kidney injury, and inflammatory markers were elevated (Table 1). Cardiology, Infectious Disease, and Rheumatology were consulted. Given the severity of symptoms and refractory shock, the patient was admitted to the paediatric ICU.

**Table 1.** Initial laboratory results on hospital admission.

	Result	Reference values
**Infectious**		
COVID-19 Ab IgG	Positive	Negative
**Cardiovascular**		
BNP	828.4 pg/ml	≤100 pg/ml
Troponin	14.927 ng/ml	< 0.029 ng/ml
D-Dimer	0.66 ug/ml FEU	0.27–0.42 ug/ml FEU
**Renal**		
BUN	20 mg/dl	6–20 mg/dl
Creatinine	1.31 mg/dl	0.62–1.09 mg/dl
**Rheumatologic**		
CRP	31.8 mg/dl	<0.5 mg/dl
ESR	19 mm/hr	0–10 mm/hr
Ferritin	662.10 ng/ml	4.4–207 ng/ml
Procalcitonin	3.3 ng/ml	0–0.15 ng/ml
LDH	340 U/L	130–250 U/L
IL-6	477.7 pg/ml	0–12.2 pg/ml
Lactate (POCT)	10.98 mmol/L	0–2.19 mmol/L

BNP: brain natriuretic peptide, BUN: blood urea nitrogen, CRP: c-reactive protein, ESR: erythrocyte sedimentation rate, LDH: lactate dehydrogenase, IL-6: interleukin-6

**Figure 1:**
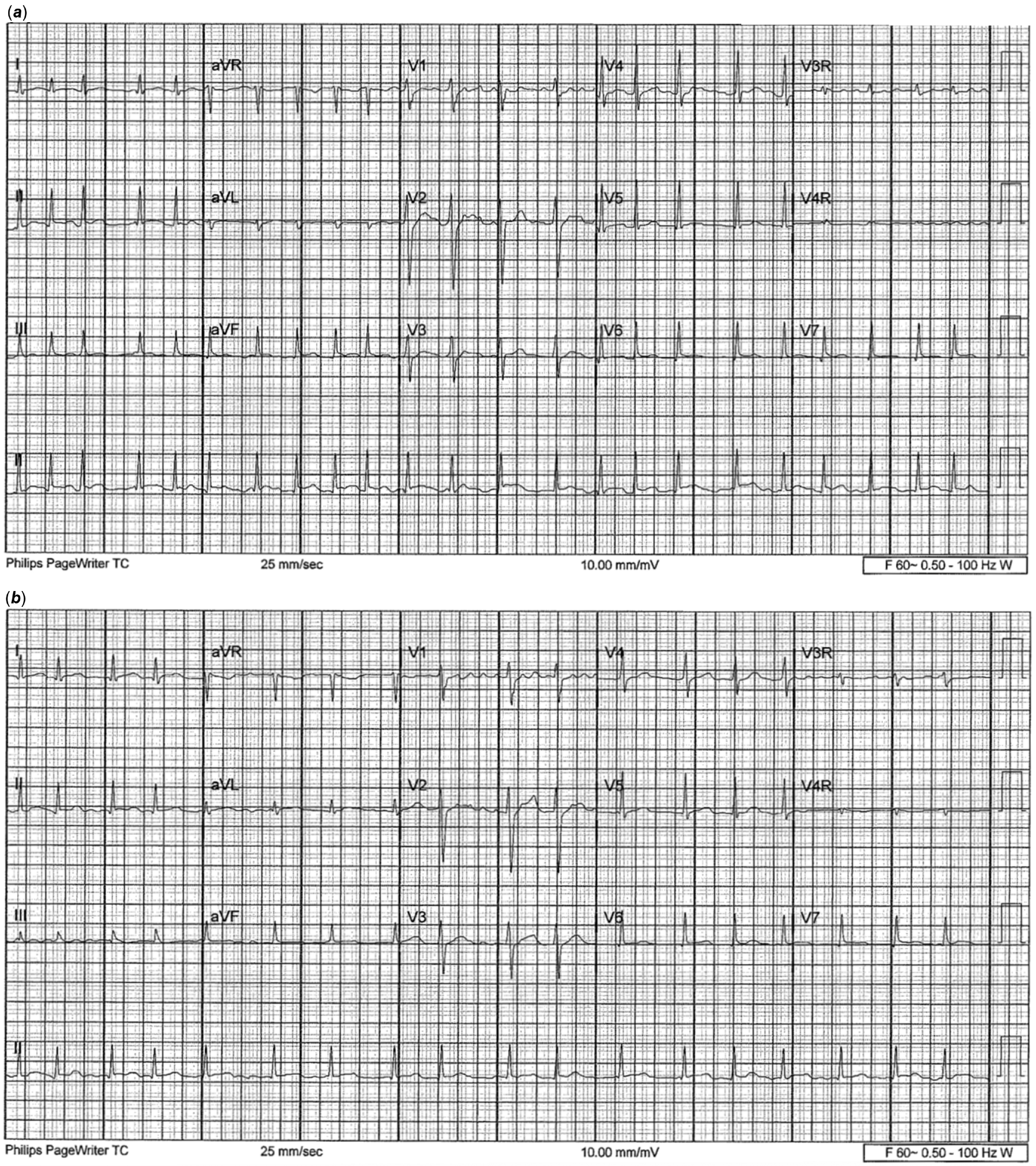
(***a***) ECG showing atrial fibrillation on hospital day 3 prior to cardioversion. (***b***) Second episode of atrial fibrillation; ECG on hospital day 4.

He was started on pulse solumedrol 30 mg/kg/day for 3 days per rheumatology recommendations, followed by tapered doses. The patient received recommended intravenous immunoglobulin treatment for multi-system inflammatory syndrome in children (100 g, weight-based maximum). Milrinone 0.5 mcg/kg/min and Aspirin 81 mg were started. Serial troponin continued to be elevated, however, down trending throughout admission. Multiple echocardiograms were obtained and significant for small pericardial effusion, but normal cardiac function, ejection fraction, and no sign of pulmonary hypertension. No coronary abnormalities were noted. On hospital day 1, the patient complained of acute chest pain without hypoxia that improved with morphine. CT chest was done and did not show evidence of pulmonary embolus. No evidence of strain on telemetry. He remained persistently hypotensive. On day 2, the patient was started on a 5-day course of biologic therapy with recombinant IL-1 antagonist Anakinra, 100 mg twice per day given the severity of presentation with elevated inflammatory markers suggesting cytokine storm. On day 3, premature atrial contractions were noted on telemetry with subsequent atrial fibrillation requiring cardioversion with 100 J and 150 J (Fig 1a). He remained stable until day 4 with new episode of atrial fibrillation requiring cardioversion with 150 J (Fig 1b) and a bolus dose of amiodarone. Low-dose epinephrine 0.02–0.05 was necessary even with sinus rhythm for refractory hypotension with orthostatic hypotension out of proportion to his clinical picture. The patient remained on sinus rhythm, but noted to have a 5 beat run of wide complex tachycardia on day 11.

Repeat labs showed improvement, with down trending inflammatory markers, troponin, and brain natriuretic peptide. The patient was transitioned to acute care floor on day 14 with the resolution of cardiac arrhythmias. He continued to have dysautonomia with orthostatic hypotension, and fludrocortisone and midodrine were started with improvement in blood pressure. Rehabilitation medicine was consulted for therapy given severe de-conditioning. He was discharged on day 16 with follow-up in Cardiology, Rheumatology and Physical/Occupational Therapy.

## Discussion

Multi-system inflammatory syndrome in children is a multi-inflammatory condition documented in children and adults that follows infection with COVID-19 meeting criteria with fever >38C, elevated inflammatory markers, and involvement of >2 organ systems.^5^ Cases are classified as mild if the patient does not require vasoactive support, presents with minimal organ injury, and requires minimal to no respiratory support; severe cases present with mild to severe organ injury and require significant respiratory support warranting ICU admission.^5^ Antibody-mediated hyper-inflammatory response has been described, supported by the positive response seen in patients treated with intravenous immunoglobulin.^2^ Similarly, macrophage activation, cardiac fibroblast, and cardiomyocyte stretching have been proposed to cause elevated interleukin-6 triggering an immunological cascade.^6^ Current guidelines for multi-system inflammatory syndrome in children treatment include starting low-dose aspirin, steroids, and intravenous immunoglobulin for all patients (dose dependent on severity) and biologic treatment with tocilizumab, anakinra, or infliximab for severe cases.^5^ In the case presented, the elevated troponin levels were likely due to cardiac muscle stress from inflammation given no evidence of coronary insufficiency or ST-segment changes on echocardiogram and electrocardiogram, respectively. Similarly, inflammatory stress and metabolic alterations may have led to the development of cardiac arrhythmia in this patient.^7^ Monitoring for cardiovascular complications via serial troponin with early intervention is essential for multi-system inflammatory syndrome in children patients.

## Conclusion

Cardiovascular manifestations of COVID-19 in children remain a topic of novel investigation as literature in this population is lacking. Emphasis has been frequently placed on the respiratory manifestations of the virus, and paediatric cardiovascular complications and treatment are not well understood. This case illustrates the potential for cardiac arrhythmias in children affected by the novel virus and prompts for further investigations and need for research regarding the rates of cardiac dysrhythmias in multi-system inflammatory syndrome in children. The case highlights the importance of developing management strategies in patients with multi-system inflammatory syndrome in children in the setting of viral infection with COVID-19. It is important that children with COVID-19 infection be screened for cardiac complications through cardiac troponin and brain natriuretic peptide levels to minimise the delay in diagnosis and treatment.^8^
